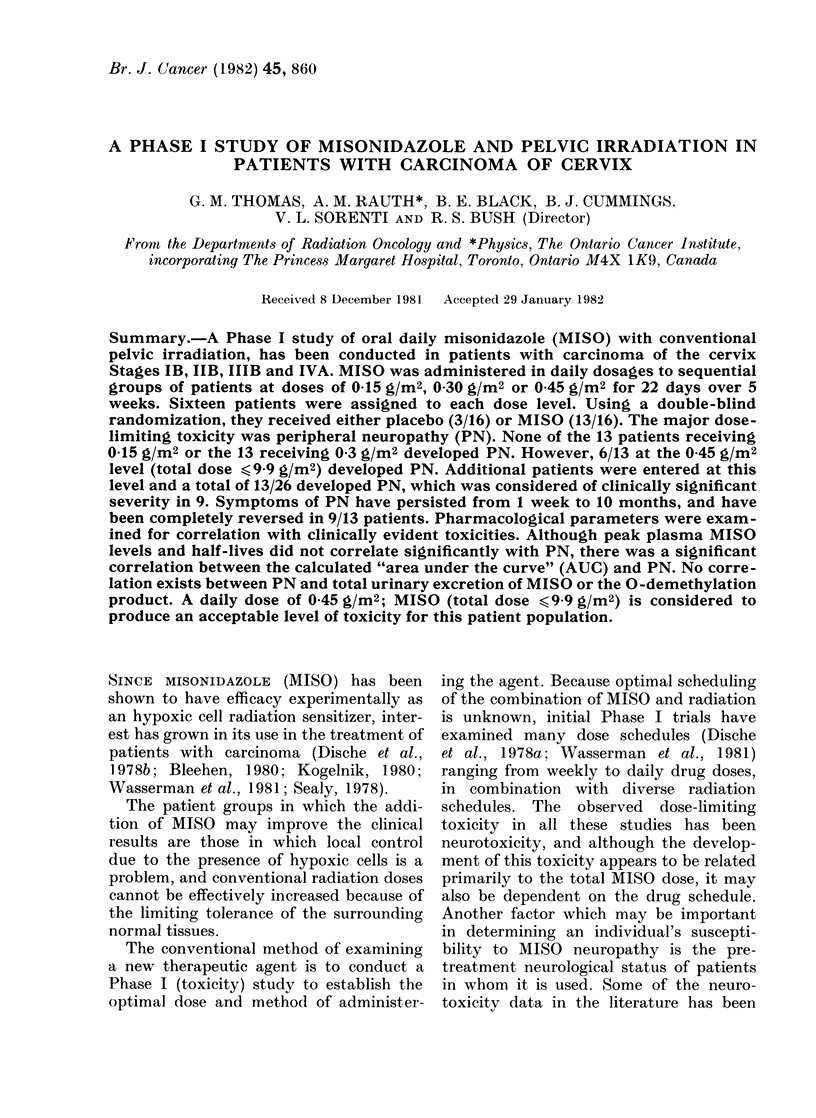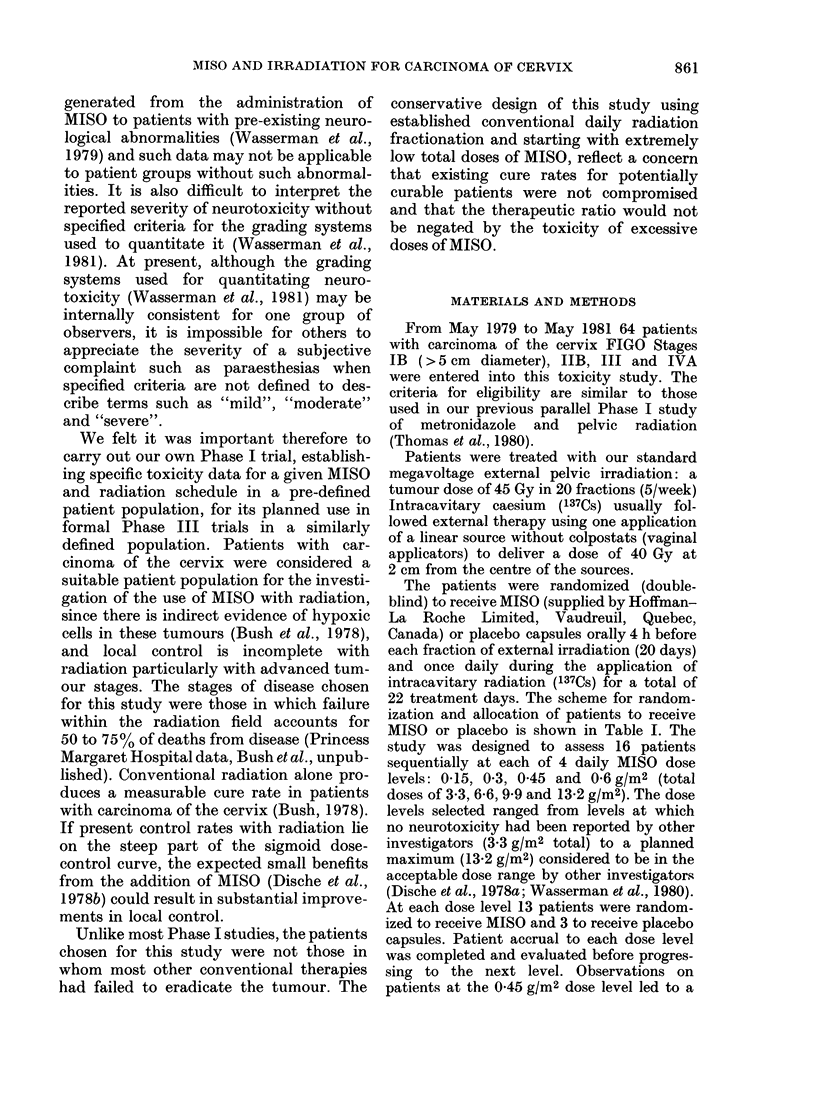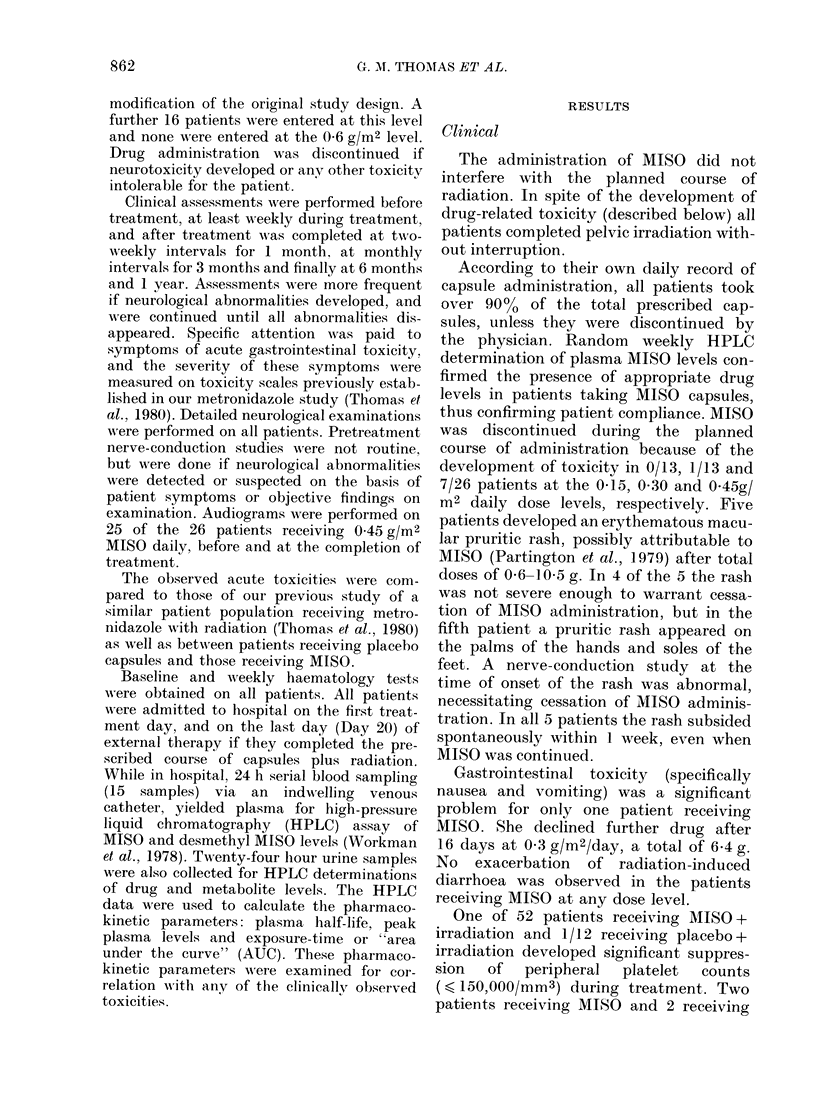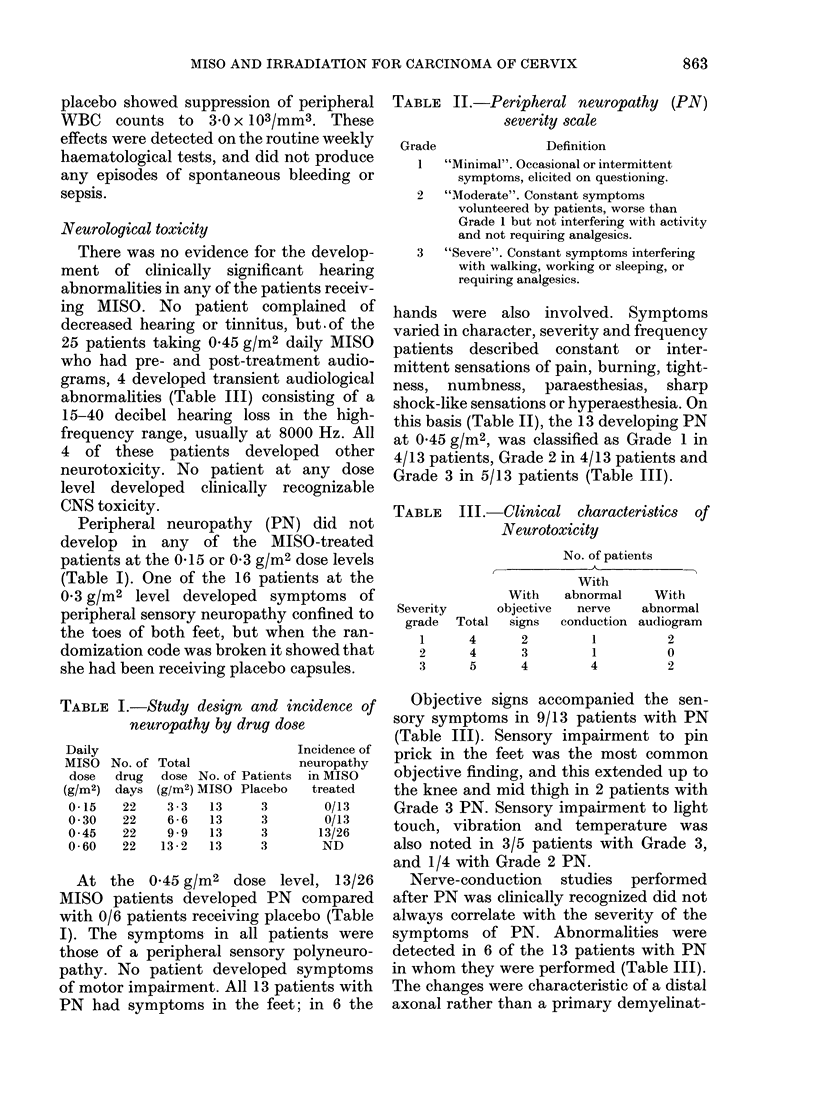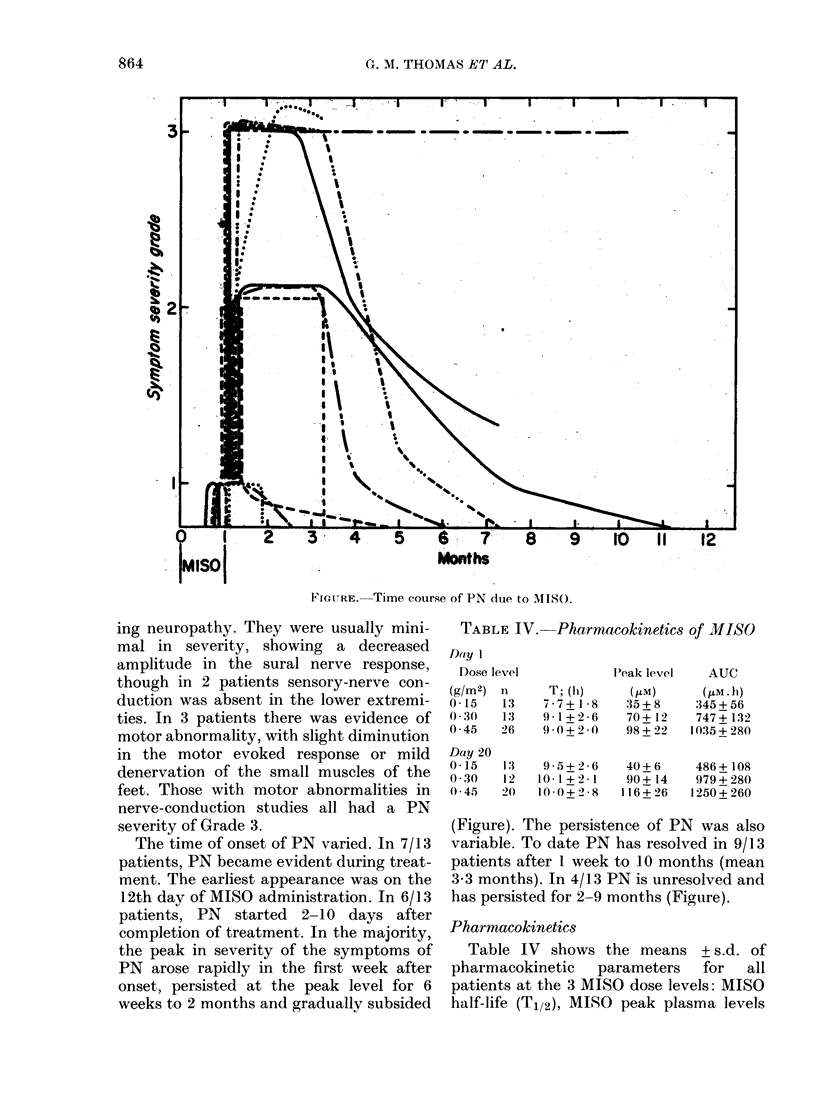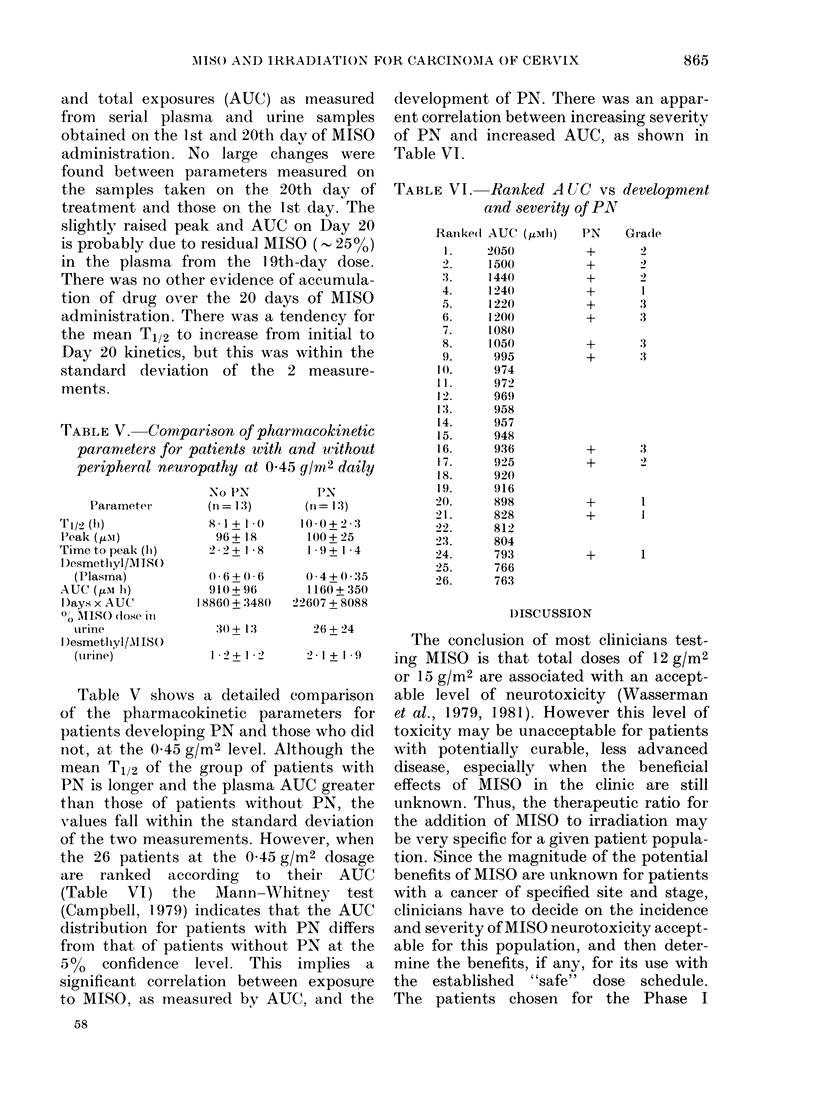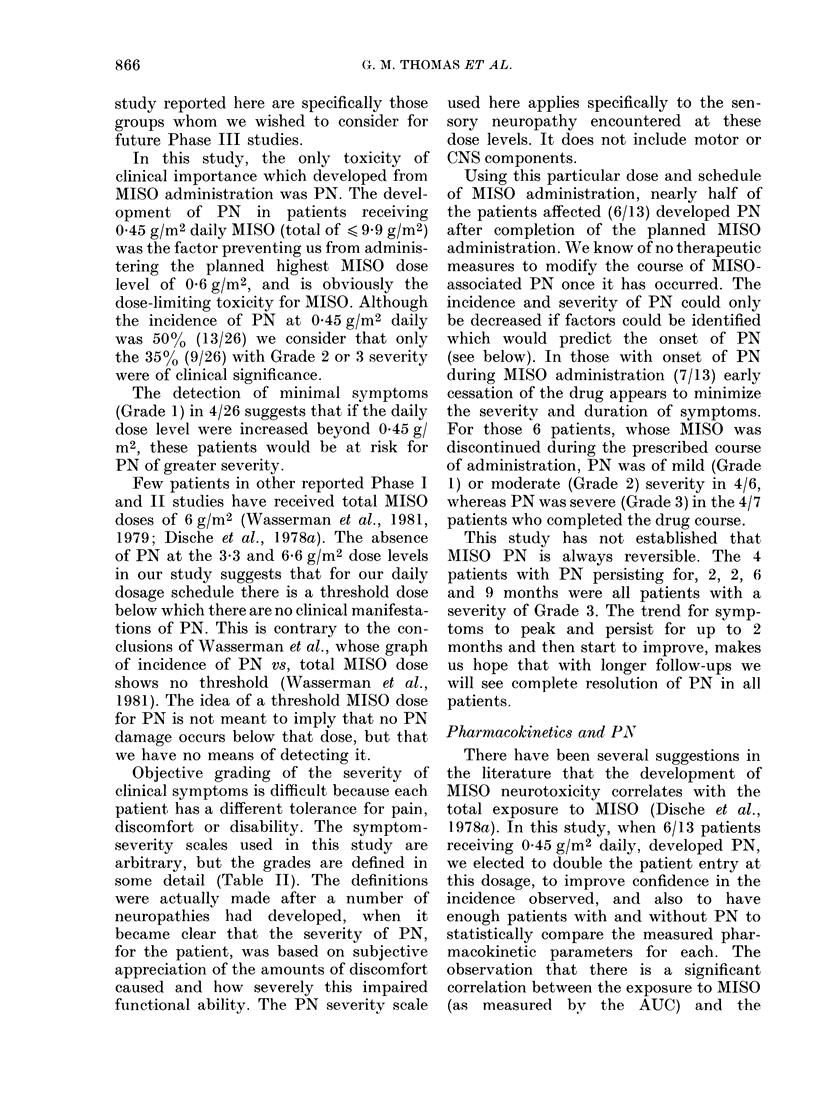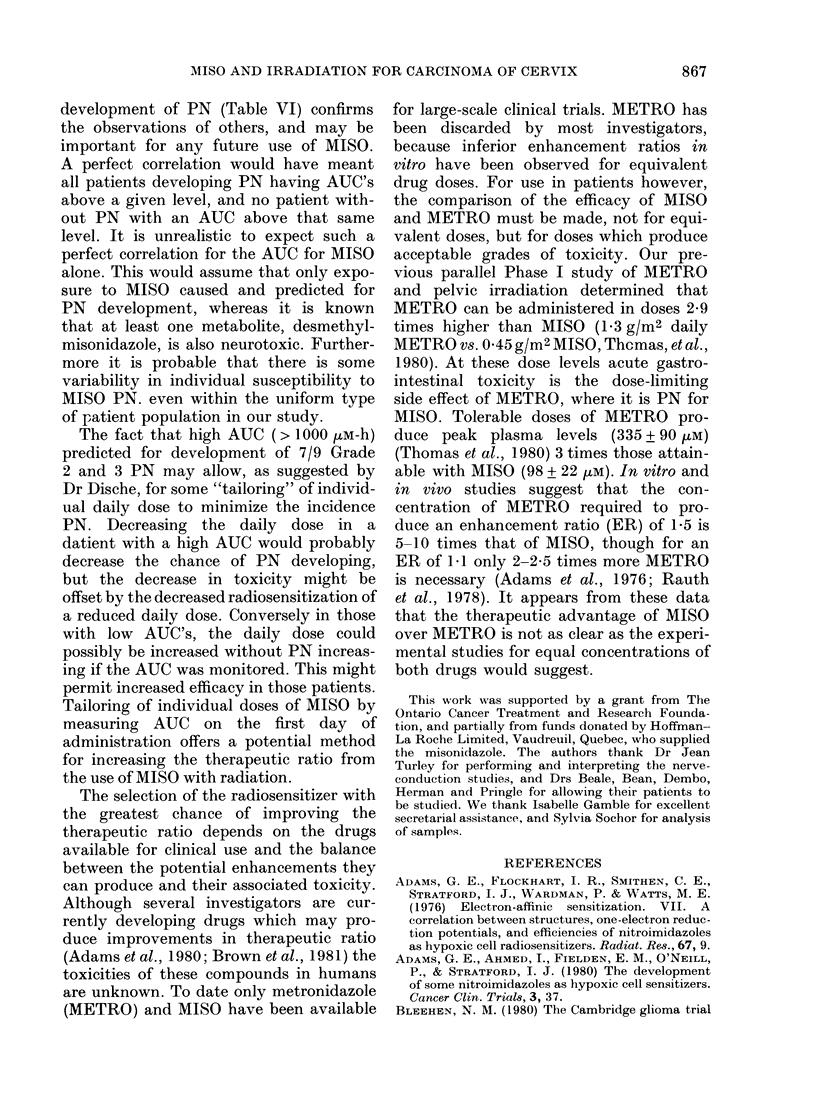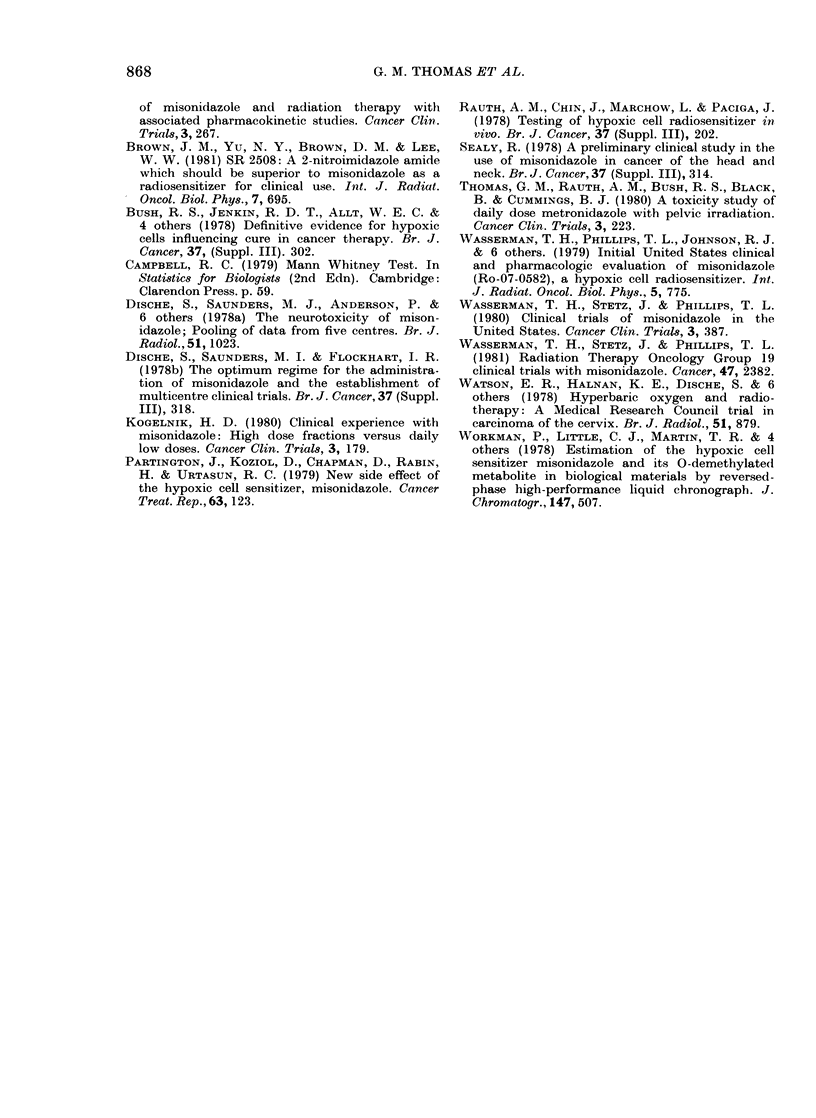# A phase I study of misonidazole and pelvic irradiation in patients with carcinoma of cervix.

**DOI:** 10.1038/bjc.1982.138

**Published:** 1982-06

**Authors:** G. M. Thomas, A. M. Rauth, B. E. Black, B. J. Cummings, V. L. Sorenti, R. S. Bush

## Abstract

A Phase I study of oral daily misonidazole (MISO) with conventional pelvic irradiation, has been conducted in patients with carcinoma of the cervix Stages IB, IIB, IIIB and IVA. MISO was administered in daily dosages to sequential groups of patients at doses of 0.15 g/m2, 0.30 g/m2 or 0.45 g/m2 for 22 days over 5 weeks. Sixteen patients were assigned to each dose level. Using a double-blind randomization, they received either placebo (3/16) or MISO (13/16). The major dose-limiting toxicity was peripheral neuropathy (PN). None of the 13 patients receiving 0.15 g/m2 or the 13 receiving 0.3 g/m2 developed PN. However, 6/13 at the 0.45 g/m2 level (total dose less than or equal to 9.9 g/m2) developed PN. Additional patients were entered at this level and a total of 13/26 developed PN, which was considered of clinically significant severity in 9. Symptoms of PN have persisted from 1 week to 10 months, and have been completely reversed in 9/13 patients. Pharmacological parameters were examined for correlation with clinically evident toxicities. Although peak plasma MISO levels and half-lives did not correlate significantly with PN, there was a significant correlation between the calculated "area under the curve" (AUC) and PN. No correlation exists between PN and total urinary excretion of MISO or the O-demethylation product. A daily dose of 0.45 g/m2; MISO (total dose less than or equal to 9.9 g/m2) is considered to produce an acceptable level of toxicity for this patient population.


*Br. J. Cancer* (1982) **45**, 860

### A PHASE I STUDY OF MISONIDAZOLE AND PELVIC IRRADIATION IN PATIENTS WITH CARCINOMA OF CERVIX

G. M. THOMAS, A. M. RAUTH*, B. E. BLACK, B. J. CUMMINGS, V. L. SORENTI AND R. S. BUSH (Director)

*From the Departments of Radiation Oncology and *Physics, The Ontario Cancer Institute, incorporating The Princess Margaret Hospital, Toronto, Ontario M4X 1K9, Canada*

Received 8 December 1981 Accepted 29 January 1982

**Summary.—A Phase I study of oral daily misonidazole (MISO) with conventional pelvic irradiation, has been conducted in patients with carcinoma of the cervix Stages IB, IIB, IIIB and IVA. MISO was administered in daily dosages to sequential groups of patients at doses of 0·15 g/m², 0·30 g/m² or 0·45 g/m² for 22 days over 5 weeks. Sixteen patients were assigned to each dose level. Using a double-blind randomization, they received either placebo (3/16) or MISO (13/16). The major dose-limiting toxicity was peripheral neuropathy (PN). None of the 13 patients receiving 0·15 g/m² or the 13 receiving 0·3 g/m² developed PN. However, 6/13 at the 0·45 g/m² level (total dose $\leqslant$9·9 g/m²) developed PN. Additional patients were entered at this level and a total of 13/26 developed PN, which was considered of clinically significant severity in 9. Symptoms of PN have persisted from 1 week to 10 months, and have been completely reversed in 9/13 patients. Pharmacological parameters were examined for correlation with clinically evident toxicities. Although peak plasma MISO levels and half-lives did not correlate significantly with PN, there was a significant correlation between the calculated "area under the curve" (AUC) and PN. No correlation exists between PN and total urinary excretion of MISO or the O-demethylation product. A daily dose of 0·45 g/m²; MISO (total dose $\leqslant$9·9 g/m²) is considered to produce an acceptable level of toxicity for this patient population.**

SINCE MISONIDAZOLE (MISO) has been shown to have efficacy experimentally as an hypoxic cell radiation sensitizer, interest has grown in its use in the treatment of patients with carcinoma (Dische *et al.*, 1978*b*; Bleehen, 1980; Kogelnik, 1980; Wasserman *et al.*, 1981; Sealy, 1978).

The patient groups in which the addition of MISO may improve the clinical results are those in which local control due to the presence of hypoxic cells is a problem, and conventional radiation doses cannot be effectively increased because of the limiting tolerance of the surrounding normal tissues.

The conventional method of examining a new therapeutic agent is to conduct a Phase I (toxicity) study to establish the optimal dose and method of administering the agent. Because optimal scheduling of the combination of MISO and radiation is unknown, initial Phase I trials have examined many dose schedules (Dische *et al.*, 1978*a*; Wasserman *et al.*, 1981) ranging from weekly to daily drug doses, in combination with diverse radiation schedules. The observed dose-limiting toxicity in all these studies has been neurotoxicity, and although the development of this toxicity appears to be related primarily to the total MISO dose, it may also be dependent on the drug schedule. Another factor which may be important in determining an individual's susceptibility to MISO neuropathy is the pretreatment neurological status of patients in whom it is used. Some of the neurotoxicity data in the literature has been generated from the administration of MISO to patients with pre-existing neurological abnormalities (Wasserman *et al.*, 1979) and such data may not be applicable to patient groups without such abnormalities. It is also difficult to interpret the reported severity of neurotoxicity without specified criteria for the grading systems used to quantitate it (Wasserman *et al.*, 1981). At present, although the grading systems used for quantitating neurotoxicity (Wasserman *et al.*, 1981) may be internally consistent for one group of observers, it is impossible for others to appreciate the severity of a subjective complaint such as paraesthesias when specified criteria are not defined to describe terms such as "mild", "moderate" and "severe".

We felt it was important therefore to carry out our own Phase I trial, establishing specific toxicity data for a given MISO and radiation schedule in a pre-defined patient population, for its planned use in formal Phase III trials in a similarly defined population. Patients with carcinoma of the cervix were considered a suitable patient population for the investigation of the use of MISO with radiation, since there is indirect evidence of hypoxic cells in these tumours (Bush *et al.*, 1978), and local control is incomplete with radiation particularly with advanced tumour stages. The stages of disease chosen for this study were those in which failure within the radiation field accounts for 50 to 75% of deaths from disease (Princess Margaret Hospital data, Bush *et al.*, unpublished). Conventional radiation alone produces a measurable cure rate in patients with carcinoma of the cervix (Bush, 1978). If present control rates with radiation lie on the steep part of the sigmoid dose-control curve, the expected small benefits from the addition of MISO (Dische *et al.*, 1978*b*) could result in substantial improvements in local control.

Unlike most Phase I studies, the patients chosen for this study were not those in whom most other conventional therapies had failed to eradicate the tumour. The conservative design of this study using established conventional daily radiation fractionation and starting with extremely low total doses of MISO, reflect a concern that existing cure rates for potentially curable patients were not compromised and that the therapeutic ratio would not be negated by the toxicity of excessive doses of MISO.

### MATERIALS AND METHODS

From May 1979 to May 1981 64 patients with carcinoma of the cervix FIGO Stages IB (>5 cm diameter), IIB, III and IVA were entered into this toxicity study. The criteria for eligibility are similar to those used in our previous parallel Phase I study of metronidazole and pelvic radiation (Thomas *et al.*, 1980).

Patients were treated with our standard megavoltage external pelvic irradiation: a tumour dose of 45 Gy in 20 fractions (5/week) Intracavitary caesium ($^{137}Cs$) usually followed external therapy using one application of a linear source without colpostats (vaginal applicators) to deliver a dose of 40 Gy at 2 cm from the centre of the sources.

The patients were randomized (double-blind) to receive MISO (supplied by Hoffman–La Roche Limited, Vaudreuil, Quebec, Canada) or placebo capsules orally 4 h before each fraction of external irradiation (20 days) and once daily during the application of intracavitary radiation ($^{137}Cs$) for a total of 22 treatment days. The scheme for randomization and allocation of patients to receive MISO or placebo is shown in Table I. The study was designed to assess 16 patients sequentially at each of 4 daily MISO dose levels: 0·15, 0·3, 0·45 and 0·6 g/m$^2$ (total doses of 3·3, 6·6, 9·9 and 13·2 g/m$^2$). The dose levels selected ranged from levels at which no neurotoxicity had been reported by other investigators (3·3 g/m$^2$ total) to a planned maximum (13·2 g/m$^2$) considered to be in the acceptable dose range by other investigators (Dische *et al.*, 1978*a*; Wasserman *et al.*, 1980). At each dose level 13 patients were randomized to receive MISO and 3 to receive placebo capsules. Patient accrual to each dose level was completed and evaluated before progressing to the next level. Observations on patients at the 0·45 g/m$^2$ dose level led to a modification of the original study design. A further 16 patients were entered at this level and none were entered at the 0·6 g/m² level. Drug administration was discontinued if neurotoxicity developed or any other toxicity intolerable for the patient.

Clinical assessments were performed before treatment, at least weekly during treatment, and after treatment was completed at two-weekly intervals for 1 month, at monthly intervals for 3 months and finally at 6 months and 1 year. Assessments were more frequent if neurological abnormalities developed, and were continued until all abnormalities disappeared. Specific attention was paid to symptoms of acute gastrointestinal toxicity, and the severity of these symptoms were measured on toxicity scales previously established in our metronidazole study (Thomas *et al.*, 1980). Detailed neurological examinations were performed on all patients. Pretreatment nerve-conduction studies were not routine, but were done if neurological abnormalities were detected or suspected on the basis of patient symptoms or objective findings on examination. Audiograms were performed on 25 of the 26 patients receiving 0·45 g/m² MISO daily, before and at the completion of treatment.

The observed acute toxicities were compared to those of our previous study of a similar patient population receiving metronidazole with radiation (Thomas *et al.*, 1980) as well as between patients receiving placebo capsules and those receiving MISO.

Baseline and weekly haematology tests were obtained on all patients. All patients were admitted to hospital on the first treatment day, and on the last day (Day 20) of external therapy if they completed the prescribed course of capsules plus radiation. While in hospital, 24 h serial blood sampling (15 samples) via an indwelling venous catheter, yielded plasma for high-pressure liquid chromatography (HPLC) assay of MISO and desmethyl MISO levels (Workman *et al.*, 1978). Twenty-four hour urine samples were also collected for HPLC determinations of drug and metabolite levels. The HPLC data were used to calculate the pharmacokinetic parameters: plasma half-life, peak plasma levels and exposure-time or "area under the curve" (AUC). These pharmacokinetic parameters were examined for correlation with any of the clinically observed toxicities.

### RESULTS

### *Clinical*

The administration of MISO did not interfere with the planned course of radiation. In spite of the development of drug-related toxicity (described below) all patients completed pelvic irradiation without interruption.

According to their own daily record of capsule administration, all patients took over 90% of the total prescribed capsules, unless they were discontinued by the physician. Random weekly HPLC determination of plasma MISO levels confirmed the presence of appropriate drug levels in patients taking MISO capsules, thus confirming patient compliance. MISO was discontinued during the planned course of administration because of the development of toxicity in 0/13, 1/13 and 7/26 patients at the 0·15, 0·30 and 0·45g/m² daily dose levels, respectively. Five patients developed an erythematous macular pruritic rash, possibly attributable to MISO (Partington *et al.*, 1979) after total doses of 0·6–10·5 g. In 4 of the 5 the rash was not severe enough to warrant cessation of MISO administration, but in the fifth patient a pruritic rash appeared on the palms of the hands and soles of the feet. A nerve-conduction study at the time of onset of the rash was abnormal, necessitating cessation of MISO administration. In all 5 patients the rash subsided spontaneously within 1 week, even when MISO was continued.

Gastrointestinal toxicity (specifically nausea and vomiting) was a significant problem for only one patient receiving MISO. She declined further drug after 16 days at 0·3 g/m²/day, a total of 6·4 g. No exacerbation of radiation-induced diarrhoea was observed in the patients receiving MISO at any dose level.

One of 52 patients receiving MISO + irradiation and 1/12 receiving placebo + irradiation developed significant suppression of peripheral platelet counts ($\leqslant$ 150,000/mm³) during treatment. Two patients receiving MISO and 2 receiving placebo showed suppression of peripheral WBC counts to $3{\cdot}0 \times 10^3/mm^3$. These effects were detected on the routine weekly haematological tests, and did not produce any episodes of spontaneous bleeding or sepsis.

*Neurological toxicity*

There was no evidence for the development of clinically significant hearing abnormalities in any of the patients receiving MISO. No patient complained of decreased hearing or tinnitus, but of the 25 patients taking 0·45 $g/m^2$ daily MISO who had pre- and post-treatment audiograms, 4 developed transient audiological abnormalities (Table III) consisting of a 15–40 decibel hearing loss in the high-frequency range, usually at 8000 Hz. All 4 of these patients developed other neurotoxicity. No patient at any dose level developed clinically recognizable CNS toxicity.

Peripheral neuropathy (PN) did not develop in any of the MISO-treated patients at the 0·15 or 0·3 $g/m^2$ dose levels (Table I). One of the 16 patients at the 0·3 $g/m^2$ level developed symptoms of peripheral sensory neuropathy confined to the toes of both feet, but when the randomization code was broken it showed that she had been receiving placebo capsules.

TABLE I.—*Study design and incidence of neuropathy by drug dose*

| Daily MISO dose ($g/m^2$) | No. of drug days | Total dose ($g/m^2$) | No. of MISO | Patients Placebo | Incidence of neuropathy in MISO treated |
|---|---|---|---|---|---|
| 0·15 | 22 | 3·3 | 13 | 3 | 0/13 |
| 0·30 | 22 | 6·6 | 13 | 3 | 0/13 |
| 0·45 | 22 | 9·9 | 13 | 3 | 13/26 |
| 0·60 | 22 | 13·2 | 13 | 3 | ND |

At the 0·45 $g/m^2$ dose level, 13/26 MISO patients developed PN compared with 0/6 patients receiving placebo (Table I). The symptoms in all patients were those of a peripheral sensory polyneuropathy. No patient developed symptoms of motor impairment. All 13 patients with PN had symptoms in the feet; in 6 the hands were also involved. Symptoms varied in character, severity and frequency patients described constant or intermittent sensations of pain, burning, tightness, numbness, paraesthesias, sharp shock-like sensations or hyperaesthesia. On this basis (Table II), the 13 developing PN at 0·45 $g/m^2$, was classified as Grade 1 in 4/13 patients, Grade 2 in 4/13 patients and Grade 3 in 5/13 patients (Table III).

TABLE II.—*Peripheral neuropathy (PN) severity scale*

| Grade | Definition |
|---|---|
| 1 | "Minimal". Occasional or intermittent symptoms, elicited on questioning. |
| 2 | "Moderate". Constant symptoms volunteered by patients, worse than Grade 1 but not interfering with activity and not requiring analgesics. |
| 3 | "Severe". Constant symptoms interfering with walking, working or sleeping, or requiring analgesics. |

TABLE III.—*Clinical characteristics of Neurotoxicity*

| | No. of patients | | | |
|---|---|---|---|---|
| Severity grade | Total | With objective signs | With abnormal nerve conduction | With abnormal audiogram |
| 1 | 4 | 2 | 1 | 2 |
| 2 | 4 | 3 | 1 | 0 |
| 3 | 5 | 4 | 4 | 2 |

Objective signs accompanied the sensory symptoms in 9/13 patients with PN (Table III). Sensory impairment to pin prick in the feet was the most common objective finding, and this extended up to the knee and mid thigh in 2 patients with Grade 3 PN. Sensory impairment to light touch, vibration and temperature was also noted in 3/5 patients with Grade 3, and 1/4 with Grade 2 PN.

Nerve-conduction studies performed after PN was clinically recognized did not always correlate with the severity of the symptoms of PN. Abnormalities were detected in 6 of the 13 patients with PN in whom they were performed (Table III). The changes were characteristic of a distal axonal rather than a primary demyelinat-

FIGURE.—Time course of PN due to MISO.

ing neuropathy. They were usually minimal in severity, showing a decreased amplitude in the sural nerve response, though in 2 patients sensory-nerve conduction was absent in the lower extremities. In 3 patients there was evidence of motor abnormality, with slight diminution in the motor evoked response or mild denervation of the small muscles of the feet. Those with motor abnormalities in nerve-conduction studies all had a PN severity of Grade 3.

The time of onset of PN varied. In 7/13 patients, PN became evident during treatment. The earliest appearance was on the 12th day of MISO administration. In 6/13 patients, PN started 2–10 days after completion of treatment. In the majority, the peak in severity of the symptoms of PN arose rapidly in the first week after onset, persisted at the peak level for 6 weeks to 2 months and gradually subsided (Figure). The persistence of PN was also variable. To date PN has resolved in 9/13 patients after 1 week to 10 months (mean 3·3 months). In 4/13 PN is unresolved and has persisted for 2–9 months (Figure).

TABLE IV.—*Pharmacokinetics of MISO*

| Dose level ($g/m^2$) | n | $T_{1/2}$ (h) | Peak level (μM) | AUC (μM.h) |
|---|---|---|---|---|
| *Day* 1 | | | | |
| 0·15 | 13 | 7·7 ± 1·8 | 35 ± 8 | 345 ± 56 |
| 0·30 | 13 | 9·1 ± 2·6 | 70 ± 12 | 747 ± 132 |
| 0·45 | 26 | 9·0 ± 2·0 | 98 ± 22 | 1035 ± 280 |
| *Day* 20 | | | | |
| 0·15 | 13 | 9·5 ± 2·6 | 40 ± 6 | 486 ± 108 |
| 0·30 | 12 | 10·1 ± 2·1 | 90 ± 14 | 979 ± 280 |
| 0·45 | 20 | 10·0 ± 2·8 | 116 ± 26 | 1250 ± 260 |

*Pharmacokinetics*

Table IV shows the means ± s.d. of pharmacokinetic parameters for all patients at the 3 MISO dose levels: MISO half-life ($T_{1/2}$), MISO peak plasma levels and total exposures (AUC) as measured from serial plasma and urine samples obtained on the 1st and 20th day of MISO administration. No large changes were found between parameters measured on the samples taken on the 20th day of treatment and those on the 1st day. The slightly raised peak and AUC on Day 20 is probably due to residual MISO ($\sim 25\%$) in the plasma from the 19th-day dose. There was no other evidence of accumulation of drug over the 20 days of MISO administration. There was a tendency for the mean $T_{1/2}$ to increase from initial to Day 20 kinetics, but this was within the standard deviation of the 2 measurements.

TABLE V.—*Comparison of pharmacokinetic parameters for patients with and without peripheral neuropathy at* 0·45 *g/m*$^2$ *daily*

| Parameter | No PN (n = 13) | PN (n = 13) |
|---|---|---|
| $T_{1/2}$ (h) | 8·1 ± 1·0 | 10·0 ± 2·3 |
| Peak (μM) | 96 ± 18 | 100 ± 25 |
| Time to peak (h) | 2·2 ± 1·8 | 1·9 ± 1·4 |
| Desmethyl/MISO (Plasma) | 0·6 ± 0·6 | 0·4 ± 0·35 |
| AUC (μM h) | 910 ± 96 | 1160 ± 350 |
| Days x AUC | 18860 ± 3480 | 22607 ± 8088 |
| % MISO dose in urine | 30 ± 13 | 26 ± 24 |
| Desmethyl/MISO (urine) | 1·2 ± 1·2 | 2·1 ± 1·9 |

Table V shows a detailed comparison of the pharmacokinetic parameters for patients developing PN and those who did not, at the 0·45 g/m$^2$ level. Although the mean $T_{1/2}$ of the group of patients with PN is longer and the plasma AUC greater than those of patients without PN, the values fall within the standard deviation of the two measurements. However, when the 26 patients at the 0·45 g/m$^2$ dosage are ranked according to their AUC (Table VI) the Mann–Whitney test (Campbell, 1979) indicates that the AUC distribution for patients with PN differs from that of patients without PN at the 5% confidence level. This implies a significant correlation between exposure to MISO, as measured by AUC, and the development of PN. There was an apparent correlation between increasing severity of PN and increased AUC, as shown in Table VI.

TABLE VI.—*Ranked AUC* vs *development and severity of PN*

| Ranked | AUC (μMh) | PN | Grade |
|---|---|---|---|
| 1. | 2050 | + | 2 |
| 2. | 1500 | + | 2 |
| 3. | 1440 | + | 2 |
| 4. | 1240 | + | 1 |
| 5. | 1220 | + | 3 |
| 6. | 1200 | + | 3 |
| 7. | 1080 | | |
| 8. | 1050 | + | 3 |
| 9. | 995 | + | 3 |
| 10. | 974 | | |
| 11. | 972 | | |
| 12. | 969 | | |
| 13. | 958 | | |
| 14. | 957 | | |
| 15. | 948 | | |
| 16. | 936 | + | 3 |
| 17. | 925 | + | 2 |
| 18. | 920 | | |
| 19. | 916 | | |
| 20. | 898 | + | 1 |
| 21. | 828 | + | 1 |
| 22. | 812 | | |
| 23. | 804 | | |
| 24. | 793 | + | 1 |
| 25. | 766 | | |
| 26. | 763 | | |

### DISCUSSION

The conclusion of most clinicians testing MISO is that total doses of 12 g/m$^2$ or 15 g/m$^2$ are associated with an acceptable level of neurotoxicity (Wasserman *et al.*, 1979, 1981). However this level of toxicity may be unacceptable for patients with potentially curable, less advanced disease, especially when the beneficial effects of MISO in the clinic are still unknown. Thus, the therapeutic ratio for the addition of MISO to irradiation may be very specific for a given patient population. Since the magnitude of the potential benefits of MISO are unknown for patients with a cancer of specified site and stage, clinicians have to decide on the incidence and severity of MISO neurotoxicity acceptable for this population, and then determine the benefits, if any, for its use with the established "safe" dose schedule. The patients chosen for the Phase I

study reported here are specifically those groups whom we wished to consider for future Phase III studies.

In this study, the only toxicity of clinical importance which developed from MISO administration was PN. The development of PN in patients receiving $0{\cdot}45\ g/m^2$ daily MISO (total of $\leqslant 9{\cdot}9\ g/m^2$) was the factor preventing us from administering the planned highest MISO dose level of $0{\cdot}6\ g/m^2$, and is obviously the dose-limiting toxicity for MISO. Although the incidence of PN at $0{\cdot}45\ g/m^2$ daily was 50% (13/26) we consider that only the 35% (9/26) with Grade 2 or 3 severity were of clinical significance.

The detection of minimal symptoms (Grade 1) in 4/26 suggests that if the daily dose level were increased beyond $0{\cdot}45\ g/m^2$, these patients would be at risk for PN of greater severity.

Few patients in other reported Phase I and II studies have received total MISO doses of $6\ g/m^2$ (Wasserman *et al.*, 1981, 1979; Dische *et al.*, 1978*a*). The absence of PN at the 3·3 and $6{\cdot}6\ g/m^2$ dose levels in our study suggests that for our daily dosage schedule there is a threshold dose below which there are no clinical manifestations of PN. This is contrary to the conclusions of Wasserman *et al.*, whose graph of incidence of PN *vs*, total MISO dose shows no threshold (Wasserman *et al.*, 1981). The idea of a threshold MISO dose for PN is not meant to imply that no PN damage occurs below that dose, but that we have no means of detecting it.

Objective grading of the severity of clinical symptoms is difficult because each patient has a different tolerance for pain, discomfort or disability. The symptom-severity scales used in this study are arbitrary, but the grades are defined in some detail (Table II). The definitions were actually made after a number of neuropathies had developed, when it became clear that the severity of PN, for the patient, was based on subjective appreciation of the amounts of discomfort caused and how severely this impaired functional ability. The PN severity scale used here applies specifically to the sensory neuropathy encountered at these dose levels. It does not include motor or CNS components.

Using this particular dose and schedule of MISO administration, nearly half of the patients affected (6/13) developed PN after completion of the planned MISO administration. We know of no therapeutic measures to modify the course of MISO-associated PN once it has occurred. The incidence and severity of PN could only be decreased if factors could be identified which would predict the onset of PN (see below). In those with onset of PN during MISO administration (7/13) early cessation of the drug appears to minimize the severity and duration of symptoms. For those 6 patients, whose MISO was discontinued during the prescribed course of administration, PN was of mild (Grade 1) or moderate (Grade 2) severity in 4/6, whereas PN was severe (Grade 3) in the 4/7 patients who completed the drug course.

This study has not established that MISO PN is always reversible. The 4 patients with PN persisting for, 2, 2, 6 and 9 months were all patients with a severity of Grade 3. The trend for symptoms to peak and persist for up to 2 months and then start to improve, makes us hope that with longer follow-ups we will see complete resolution of PN in all patients.

*Pharmacokinetics and PN*

There have been several suggestions in the literature that the development of MISO neurotoxicity correlates with the total exposure to MISO (Dische *et al.*, 1978*a*). In this study, when 6/13 patients receiving $0{\cdot}45\ g/m^2$ daily, developed PN, we elected to double the patient entry at this dosage, to improve confidence in the incidence observed, and also to have enough patients with and without PN to statistically compare the measured pharmacokinetic parameters for each. The observation that there is a significant correlation between the exposure to MISO (as measured by the AUC) and the development of PN (Table VI) confirms the observations of others, and may be important for any future use of MISO. A perfect correlation would have meant all patients developing PN having AUC's above a given level, and no patient without PN with an AUC above that same level. It is unrealistic to expect such a perfect correlation for the AUC for MISO alone. This would assume that only exposure to MISO caused and predicted for PN development, whereas it is known that at least one metabolite, desmethylmisonidazole, is also neurotoxic. Furthermore it is probable that there is some variability in individual susceptibility to MISO PN. even within the uniform type of patient population in our study.

The fact that high AUC ($>1000$ μM-h) predicted for development of 7/9 Grade 2 and 3 PN may allow, as suggested by Dr Dische, for some "tailoring" of individual daily dose to minimize the incidence PN. Decreasing the daily dose in a datient with a high AUC would probably decrease the chance of PN developing, but the decrease in toxicity might be offset by the decreased radiosensitization of a reduced daily dose. Conversely in those with low AUC's, the daily dose could possibly be increased without PN increasing if the AUC was monitored. This might permit increased efficacy in those patients. Tailoring of individual doses of MISO by measuring AUC on the first day of administration offers a potential method for increasing the therapeutic ratio from the use of MISO with radiation.

The selection of the radiosensitizer with the greatest chance of improving the therapeutic ratio depends on the drugs available for clinical use and the balance between the potential enhancements they can produce and their associated toxicity. Although several investigators are currently developing drugs which may produce improvements in therapeutic ratio (Adams *et al.*, 1980; Brown *et al.*, 1981) the toxicities of these compounds in humans are unknown. To date only metronidazole (METRO) and MISO have been available for large-scale clinical trials. METRO has been discarded by most investigators, because inferior enhancement ratios *in vitro* have been observed for equivalent drug doses. For use in patients however, the comparison of the efficacy of MISO and METRO must be made, not for equivalent doses, but for doses which produce acceptable grades of toxicity. Our previous parallel Phase I study of METRO and pelvic irradiation determined that METRO can be administered in doses 2·9 times higher than MISO (1·3 g/m$^2$ daily METRO *vs.* 0·45 g/m$^2$ MISO, Thcmas, *et al.*, 1980). At these dose levels acute gastrointestinal toxicity is the dose-limiting side effect of METRO, where it is PN for MISO. Tolerable doses of METRO produce peak plasma levels ($335 \pm 90$ μM) (Thomas *et al.*, 1980) 3 times those attainable with MISO ($98 \pm 22$ μM). *In vitro* and *in vivo* studies suggest that the concentration of METRO required to produce an enhancement ratio (ER) of 1·5 is 5–10 times that of MISO, though for an ER of 1·1 only 2–2·5 times more METRO is necessary (Adams *et al.*, 1976; Rauth *et al.*, 1978). It appears from these data that the therapeutic advantage of MISO over METRO is not as clear as the experimental studies for equal concentrations of both drugs would suggest.

This work was supported by a grant from The Ontario Cancer Treatment and Research Foundation, and partially from funds donated by Hoffman–La Roche Limited, Vaudreuil, Quebec, who supplied the misonidazole. The authors thank Dr Jean Turley for performing and interpreting the nerve-conduction studies, and Drs Beale, Bean, Dembo, Herman and Pringle for allowing their patients to be studied. We thank Isabelle Gamble for excellent secretarial assistance, and Sylvia Sochor for analysis of samples.

### REFERENCES

Adams, G. E., Flockhart, I. R., Smithen, C. E., Stratford, I. J., Wardman, P. & Watts, M. E. (1976) Electron-affinic sensitization. VII. A correlation between structures, one-electron reduction potentials, and efficiencies of nitroimidazoles as hypoxic cell radiosensitizers. *Radiat. Res.*, **67**, 9.

Adams, G. E., Ahmed, I., Fielden, E. M., O'Neill, P., & Stratford, I. J. (1980) The development of some nitroimidazoles as hypoxic cell sensitizers. *Cancer Clin. Trials*, **3**, 37.

Bleehen, N. M. (1980) The Cambridge glioma trial of misonidazole and radiation therapy with associated pharmacokinetic studies. *Cancer Clin. Trials*, **3**, 267.

BROWN, J. M., YU, N. Y., BROWN, D. M. & LEE, W. W. (1981) SR 2508: A 2-nitroimidazole amide which should be superior to misonidazole as a radiosensitizer for clinical use. *Int. J. Radiat. Oncol. Biol. Phys.*, **7**, 695.

BUSH, R. S., JENKIN, R. D. T., ALLT, W. E. C. & 4 others (1978) Definitive evidence for hypoxic cells influencing cure in cancer therapy. *Br. J. Cancer*, **37**, (Suppl. III). 302.

CAMPBELL, R. C. (1979) Mann Whitney Test. In *Statistics for Biologists* (2nd Edn). Cambridge: Clarendon Press. p. 59.

DISCHE, S., SAUNDERS, M. J., ANDERSON, P. & 6 others (1978a) The neurotoxicity of misonidazole; Pooling of data from five centres. *Br. J. Radiol.*, **51**, 1023.

DISCHE, S., SAUNDERS, M. I. & FLOCKHART, I. R. (1978b) The optimum regime for the administration of misonidazole and the establishment of multicentre clinical trials. *Br. J. Cancer*, **37** (Suppl. III), 318.

KOGELNIK, H. D. (1980) Clinical experience with misonidazole: High dose fractions versus daily low doses. *Cancer Clin. Trials*, **3**, 179.

PARTINGTON, J., KOZIOL, D., CHAPMAN, D., RABIN, H. & URTASUN, R. C. (1979) New side effect of the hypoxic cell sensitizer, misonidazole. *Cancer Treat. Rep.*, **63**, 123.

RAUTH, A. M., CHIN, J., MARCHOW, L. & PACIGA, J. (1978) Testing of hypoxic cell radiosensitizer *in vivo*. *Br. J. Cancer*, **37** (Suppl. III), 202.

SEALY, R. (1978) A preliminary clinical study in the use of misonidazole in cancer of the head and neck. *Br. J. Cancer*, **37** (Suppl. III), 314.

THOMAS, G. M., RAUTH, A. M., BUSH, R. S., BLACK, B. & CUMMINGS, B. J. (1980) A toxicity study of daily dose metronidazole with pelvic irradiation. *Cancer Clin. Trials*, **3**, 223.

WASSERMAN, T. H., PHILLIPS, T. L., JOHNSON, R. J. & 6 others. (1979) Initial United States clinical and pharmacologic evaluation of misonidazole (Ro-07-0582), a hypoxic cell radiosensitizer. *Int. J. Radiat. Oncol. Biol. Phys.*, **5**, 775.

WASSERMAN, T. H., STETZ, J. & PHILLIPS, T. L. (1980) Clinical trials of misonidazole in the United States. *Cancer Clin. Trials*, **3**, 387.

WASSERMAN, T. H., STETZ, J. & PHILLIPS, T. L. (1981) Radiation Therapy Oncology Group 19 clinical trials with misonidazole. *Cancer*, **47**, 2382.

WATSON, E. R., HALNAN, K. E., DISCHE, S. & 6 others (1978) Hyperbaric oxygen and radiotherapy: A Medical Research Council trial in carcinoma of the cervix. *Br. J. Radiol.*, **51**, 879.

WORKMAN, P., LITTLE, C. J., MARTIN, T. R. & 4 others (1978) Estimation of the hypoxic cell sensitizer misonidazole and its O-demethylated metabolite in biological materials by reversed-phase high-performance liquid chronograph. *J. Chromatogr.*, **147**, 507.